# The management of dental caries in primary teeth - involving service providers and users in the design of a trial

**DOI:** 10.1186/1745-6215-13-143

**Published:** 2012-08-22

**Authors:** Zoe Marshman, Nicola Innes, Chris Deery, Melanie Hall, Chris Speed, Gail Douglas, Jan Clarkson, Helen Rodd

**Affiliations:** 1 School of Clinical Dentistry, Claremont Crescent, Sheffield, S10 2TA, UK; 2 School of Dentistry, Park Place, Dundee, DD1 4HN, UK; 3 Newcastle Clinical Trials Unit 4th Floor, William Leech Building, Framlington Place, Newcastle upon Tyne, NE2 4HH, UK; 4 Leeds Dental Institute, Clarendon Way, Leeds, LS2 9LU, UK; 5 Dental Health Services Research Unit, School of Dentistry, Park Place, Dundee, DD1 4HN, UK

**Keywords:** User involvement, Feasibility, Randomized controlled trial, Dental, Children

## Abstract

**Background:**

There is a lack of evidence for the effective management of dental caries in children’s primary teeth. The trial entitled ‘Filling Children’s Teeth: Indicated Or Not?’ (FiCTION) was designed to examine the clinical and cost effectiveness, in primary dental care, of three different approaches to the management of caries in primary teeth. However, before the FiCTION main trial commenced, a pilot trial was designed. Service provider (dentists and other members of the team including dental nurses and practice managers) and participant (child participants and their parents) involvement was incorporated into the pilot trial. The aim of this study is to describe service providers’ and users’ perspectives on the pilot trial to identify improvements to the conduct and design of the FiCTION main trial.

**Methods:**

Qualitative interviews (individual and group) were held with dentists, dental team members, children and parents involved in the FiCTION pilot trial. Individual interviews were held with four dentists and a group interview was held with 17 dental team members. Face-to-face interviews were held with four parents and children (four- to eight-years old) representing the three arms of the trial and five telephone interviews were conducted with parents. All interviews were transcribed verbatim. Framework analysis was used.

**Results:**

Overall, service providers, children and parents found the pilot trial to be well conducted and an interesting experience. Service providers highlighted the challenges of adhering to research protocols, especially managing the documentation and undertaking new clinical techniques. They indicated that the time and financial commitments were greater than they had anticipated. Particular difficulties were found recruiting suitable patients within the timeframe. For parents recruitment was apparently more related to trusting their dentist than the content of information packs. While some of the older children understood what a study was, others did not understand or were not aware they were enrolled.

**Conclusions:**

The findings provided valuable recommendations to improve the method of recruitment of dental practices and patients, the timing and content of the training, the type of support dentists would value and ways to further engage children and parents in the FiCTION main trial.

**Trial registration:**

ISRCTN77044005

## Background

Contemporary approaches advocate user involvement in research involving participants throughout the design, conduct and reporting of the research process [1]. Indeed, evidence of patient and public involvement is a mandatory requirement of many ethical committees worldwide and funding bodies including the US National Institutes for Health and the UK National Institute for Health Research. Many benefits of user involvement in research have been described; these include research which is more relevant to the needs of patients [2], improved prioritization of research areas to optimize patient benefit [3], studies that are more appropriately designed, particularly in terms of outcome measures, better recruitment [4,5] and enhanced implementation of the research findings [1]. Other benefits of involving service providers in research, particularly in feasibility studies for clinical trials, include identification of potential barriers to recruitment of both clinicians and patients [6], the management of patients’ and providers’ intervention preferences and identification of resources required to ensure thatthe trial runs smoothly and in a timely fashion [4].

To date, there is little evidence of involvement of patients and the public in the design, conduct or dissemination of dental research. A recent study that sought to identify potential factors that might contribute to parent participation in a gene bank for patients with orofacial clefts found that reassurance from trusted healthcare professionals about the purpose of the project was particularly important [7]. Another qualitative study of primary care dentist and dental care professionals’ involvement in prospective studies and clinical trials cited cultural, medico-legal and commercial concerns that acted as barriers to their involvement. Several specific difficulties raised have been ethical concerns about clinical equipoise, time commitment [8] and recruitment of suitable patients [8,9]. However, the involvement of service providers and users to contribute to the design of a dental trial has not previously been described.

Within the UK, the lack of evidence for the effective management of dental caries (decay) in children’s primary teeth causes considerable uncertainty for the dental profession and patients. Current clinical guidelines produced by the British Society of Paediatric Dentistry [10,11] are largely based on evidence for the effectiveness of restorations obtained from studies conducted in either a secondary care or specialist pediatric practice setting. While both the volume and quality of the research on which the guidance is based is limited, it is acknowledged that restorations provided in specialist clinical environments can be effective [12]. However, it is the generalizability of this evidence to a primary care setting which is in question, particularly as the majority of dental care for children is provided in primary care by general dental practitioners (GDPs).

To address this lack of evidence, a multi-center team sought funding from the National Institute for Health Research to undertake a randomized controlled trial (RCT), the findings of which will have considerable national and international impact. The trial entitled ‘Filling Children’s Teeth: Indicated Or Not?’ (FiCTION) is a three arm parallel design RCT which examines the relative clinical and cost effectiveness, in the primary care dental practice setting, of three different approaches to the management of caries in primary teeth. The three arms are:

•surgical’: based on the traditional ‘drill and fill’ approach where local anesthesia is given, caries is completely removed and a restorative material (filling or crown) is placed;

•biological’: an intermediate treatment where strategies to seal-in caries are used to stop its progression, including the use of Hall Technique preformed metal crowns;

•prevention alone’, a best practice preventive program (which is also incorporated into the other two arms) [13].

Before the FiCTION main trial commenced, a pilot trial was carried out to ensure the main trial was practicable, particularly in terms of patient recruitment/retention rates and collection of data on clinical outcomes. Service providers’ (dentists and other members of the team including dental nurses and practice managers) and participants’ (child participants and their parents) involvement was incorporated into the pilot trial. Very few clinical trials involving children have been conducted in the primary dental care setting in the UK or worldwide. The findings of the pilot RCT were then used to refine the design and conduct of the main trial.

The aim of this study is to describe service providers’ and users’ perspectives on the FiCTION pilot trial to improve the design and conduct of the FiCTION main trial. This paper reports on these perspectives and the resultant changes to the FiCTION main trial.

## Methods

Before describing the method used for user involvement, the FiCTION pilot trial will briefly be described.

### The FiCTION pilot trial

The pilot trial was conducted in three areas in the UK involving 11 practices (20 dentists) working in purposively selected primary care dental practices. The selected practices either had previous research experience or had expressed a preference in taking part in this trial. The overall target for recruitment was 200 children, with each dentist expected to recruit around 15 patients. Dentists were asked to send project information sheets to potential participants, within the correct age range (three to seven years), at the time of their recall appointment. At this appointment, children assessed as eligible were invited to participate and consent was obtained. Questionnaires were completed at this visit and also at follow-up appointments. Randomization was conducted through a web-based facility run by the clinical trials unit. The allocated treatment was provided and reviewed after three to six months. Despite an 80% uptake rate for eligible participants, at the end of the pilot trial less than 50% of the expected number of patients had been recruited.

### Service provider and user involvement

A qualitative approach was taken in order to capture the breadth of participants’ experiences and perceptions [14]. Qualitative interviews were held with dentists, dental team members, children and parents involved in the FiCTION pilot trial in two of the three sites for practical reasons. Interviews were conducted by members of the research team experienced in conducting research with children (ZM and MH).

Twenty-one dentists and dental team members representing nine practices were recruited for this study by a member of the research team (ZM). Interviews with dentists and dental team members were carried out in groups or individually at a time and venue convenient to them, either in their dental practices, in the local dental school or at the dentists’ homes. A combination of individual and group interviews was chosen to gain both experiences of individuals, but also interaction between participants and to allow participants a choice of their preferred and the most convenient method. Data saturation was achieved after individual interviews were held with four dentists (from three different practices) and a group interview was conducted with an additional 17 dental team members (ten dentists, two nurses and two practice managers) representing a further six practices. The group interview was held during one of the regular meetings about the trial, this approach was requested by the service providers to optimize the use of their time. Every member of the group was encouraged to participate in the discussion by the interviewer and contributions were made by all. All those service providers who were approached agreed to participate. The initial report of the analysis was sent back to the participants to confirm the interpretation of the data [15].

Parents and children were recruited by dentists taking part in the trial. The participants were purposively selected to ensure the three arms of the trial were represented. Interviews were conducted with parents and children (four- to eight-years old) in their own homes. Interviews were chosen as the most appropriate qualitative method for parents and children to gain their individual experiences and perceptions. Parents and children were asked if they preferred to be interviewed together or separately and in all cases the interviews were conducted separately but with the parent present in the room while their child was interviewed. Input from the parent during their child interview occurred occasionally as they felt appropriate. Additional telephone interviews were conducted with parents who expressed a desire to be contacted for discussions about the study and attempts were made to contact parents who had declined to take part in the trial, but none were available for discussion. Parents who agreed to be contacted were interviewed. All five parents approached agreed to participate but one family later withdrew due to personal circumstances. Face-to-face interviews were therefore held with four parents and children (four- to eight-years old) representing the three arms of the trial. Telephone interviews were also conducted with five additional parents who expressed a desire to be contacted for discussions about the study. Data saturation was achieved for parents but not for children.

The topic guides used to inform the interviews were derived from the areas to be assessed in the pilot trial and discussions with the trial management group. However, the specific wording of the questions was tailored to the participants [16]. A flexible approach was taken to ensure participants were encouraged to introduce their own topics about the design and conduct of the trial and any contradictory views probed.

Written consent was obtained. Pseudonyms were used to ensure confidentiality. Ethical approval was received from NHS Forth Valley Research Ethics Committee (NRES reference 10/S1402/8) and research governance approval was obtained. The registration number for the FiCTION pilot trial is ISRCTN77044005.

### Analysis

All interviews were audio-recorded and transcribed verbatim with transcription carried out as soon as possible after the interviews to allow the data to be analyzed as it was collected. The transcripts were proofed against the audio-recorded material. To allow large volumes of data to be handled while staying true to the participants’ accounts, framework analysis was conducted [17]. To improve external validity, three researchers were involved in the analysis and interpretation of the data; these researchers had differing backgrounds (dental public health, sociology and pediatric dentistry) and disagreements between researchers were resolved through discussion [15].

Framework analysis [17] involves six stages: 1) identifying initial themes, 2) labeling the data, 3) sorting the data by theme, 4) synthesizing the data, 5) developing descriptive accounts and 6) exploring explanatory accounts. First, recurring themes were identified and developed. Then the themes were grouped into a number of main and sub-themes. At this stage an initial thematic framework was created. Labels were then attached to each section of the transcripts which is called indexing; the labels represented the theme to which it was associated. The thematic framework was then further developed to add any new themes. The next step was sorting the data by theme where sections of data with the same label were brought together. Charts were then created for each of the main themes using the context and language found in the data. The entire transcripts were also reread regularly to minimize fragmentation. The nature and content of each theme was described and discussed between the researchers. Explanation of links between themes were developed and further discussed.

## Results

The results are presented as the themes that emerged from the data rather than preconceived ideas. Quotes are used to support and illustrate points. The main themes concerned:

1. involvement in the trial

2. recruitment of patients

3. training of the dental teams

4. treatments involved in the three arms of the trial

5. trial documentation

The results for dental teams, child and parents will be described within these five themes where applicable (Table 1).

**Table 1:** Themes and subthemes from dental teams, parents and children

**Themes**	**Perspective**	**Subtheme**
Involvement in the trial	Dental teams	Overall experiences
		Time and financial implications
		Whole team involvement
	Children	
	Parents	
Recruitment of patients	Dental teams Parents	
		
Training	Dental teams	
Treatments involved in the three arms of the trial	Dental teams	Preparedness
		Parent and child preferences
	Parents	
Trial documentation	Dental teams	

## Involvement in the trial

### Dental teams

Within this theme there were three subthemes of the dental team’s experiences of involvement in the trial: their overall experiences, time and financial implications and the need for whole team involvement.

#### Overall experiences

Overall, the dental teams involved found the pilot trial to be well conducted and a valuable experience. They felt the research question was of high importance and were able to describe improvements that should be made to the design and conduct of the main FiCTION trial. Participants, many of whom had no experience with research, found being involved in a project of this nature interesting and challenging. Aspects such as the academic language used, the rigid requirements of following a research protocol and communicating the research project to patients were highlighted as new experiences for many dentists and dental team members.

#### Time and financial implications

The time and money involved for dental practices taking part was mentioned as an important factor, dental team members were unclear about both these aspects before they started the trial:

"Dentist F: ‘*To be honest, being asked to do something like this, there is a funding implication for the time that it takes.... we’re primary care where the data is but it costs and if you are going to take time off to do all of the paperwork, there’s a cost to the practice and I think that puts a lot of people off, it does take a lot of time.’*"

In addition to staff time, other specific costs were mentioned such as postage; low eligibility rates for recruitment meant that many more information packs had to be sent out than had been anticipated.

#### Whole team involvement

Bearing in mind the difficulties with recruitment and the time/cost implications, the need for involvement of the whole dental team was stressed. Receptionists were viewed as key personnel to deal with patient queries and administration. Dentists preferred to have other colleagues in their practice involved to share the workload. Obtaining approval from the practice principal, as the business owner, was felt to be important.

### Children’s involvement in the trial

Despite their young age, children were able to describe their perspectives on being involved in the study. While some of the older children understood what a study was, others did not understand and were not aware, or did not remember, that they were enrolled. Those who were aware of their involvement enjoyed being asked to sign their names on the consent form and were familiar with the smiley face response format used in questionnaires as illustrated by this interchange between one boy and his mum:

"Child 1, boy aged 6 years: ‘*Yeah and I wrote my name’.*"

"Parent 1, mother: ‘*You signed them. There were circles and smiley faces…There were happy faces weren’t there? And sad faces’*"

"Child 1, boy aged 6 years: *‘And normal’.*"

Specific recommendations mentioned by children themselves to improve children’s involvement in the main trial related to the use of a cartoon character on study documentation, particularly a superhero, the introduction of opportunities for coloring and the availability of trial stickers.

### Parents’ involvement in the trial

Overall, parents’ experiences were positive and the research question was felt to be important. Parents were happy to be involved if it had minimal impact on their child but would lead to improved treatment for children in the future:

"Parent 3, mother: *‘I think that advancements in dentistry are important and that it is important to help. I wasn’t going to put him in any danger or anything. I was happy with it, he hadn’t complained and the problem was solved.’*"

The only impact described by parents was that the first check-up appointment lasted longer than usual. Some parents stated that they would have liked more updates on their child’s progress in the trial and information about the trial as a whole.

Parents also described their perceptions about their child’s involvement in the study generally, about the questionnaires and ways that children could be more actively engaged. A range of perceptions from parents about their child’s involvement were expressed. Some parents felt their child was excited to participate and felt important to be part of a study, others felt their child did not understand what the study entailed and felt dentists should spend more time talking to children about it.

"Parent 2, father*: ‘Simon was happy to help, he’s so inquisitive, he was interested. We read the letter together and he was happy to be in it and he was excited about his first filling’*."

A range of perceptions were also described about children’s understanding of the questionnaires:

"Parent 4, mother: *‘I don’t think they really understand. I asked James the questions and he said ‘yes’ to everything’*."

"Parent 3, mother: *‘I think a few things need fine tuning… it felt a bit repetitive on the kids’ one, they are the same questions…were you worried? Were you worried?’*"

This range of perceptions may have related to the age or levels of abilities of the children. A suggestion to improve the questionnaire included more pictures to supplement the text. Finally, parents recommended that the use of a cartoon character for children to relate to, material on a website for children to download and thank-you gifts (for example a toothbrushing kit) would improve children’s engagement with the trial.

## Recruitment of patients

### Dental teams’ perspectives on the recruitment of patients

Dentists described their frustration and surprise at how difficult it proved to recruit suitable patients, at least initially. The main difficulty appeared to be that the parents of the eligible children on the dentists’ lists did not respond to receiving the study information packs through the post:

"Dentist G: ‘*I think it would have gone brilliantly if we could have got a higher recruitment rate.......the ones that have bothered to look at it (the patient information pack), you look in the kids mouth and they’ve got no carious teeth and the ones that, I flag it up on the notes, they come in for a check up. I say “I sent you some information out about the trial”, “‘oh yeah, I binned it”’. That’s the overriding problem.’*"

"Dentist I: *‘Cold calling as it is technically with bumpf that lands on the doorstep! The parents round here, there’s a lot of illiteracy around here and some of the parents, they’re just not going to (respond).’*"

Dental team members also found that parents preferred appointments after school or during school holidays. This had an impact on the dentists’ opportunities to recruit patients in the required time period and also how quickly they could finish the patient’s treatment. The length of the time it takes to carry out the treatment and paperwork prevented the practice being able to ‘*squeeze*’ the trial patients into their clinical schedule.

"Practice manager E: *‘We’re only open until five, so patients who come in and want after school, they are waiting a few weeks for their treatment just because we’re full, so that slows down our work.’*"

Dedicated sessions during school holidays or weekends were recommended for the recruitment and treatment of patients in the main trial.

### Parents’ perspectives on recruitment

Parents described either receiving an information pack about the study with their child’s check-up appointment or being recruited directly at the practice. While parents felt the format and content of the information pack was generally appropriate, it was the explanations and reassurances provided by the dentists, who were evidently highly trusted, which parents felt were important in their decisions to take part:

"Parent 5, mother: ‘*It was fine, it was clear and the dentist was really nice. To be honest, I hadn’t taken much notice of the letter so it was good to have it all explained...........It was the way she explained it rather than the letter that made us want to take part’*."

The important contribution of the dentists’ explanation to recruitment was a recurring theme and is significant for the design of the main trial.

## Training

In general, the training for the pilot trial was found to be appropriate. However, for some dental teams the time between the training and commencement of recruitment was felt to be too long and specific aspects of the training could have been improved.

The timing of the training was felt to be crucial in terms of dental teams remembering what to do regarding the clinical protocol and patient documentation:

"Dentist B: ‘*It had been so long between us having training and getting started, because when we first had the training, I think we were all very clear on what the arms were but I think it was nearly 18 months was it?’*"

A dental team who had experienced a shorter time frame between training and commencing recruitment and who received training in their practice described different experiences:

"Dentist H: ‘*Doing this short, intensive training course and getting on with it actually worked fine .....we only had a couple of meetings out of our time and we got a very good way of doing it.’*"

In addition to comments about the importance of the timing of the training, other suggested ways to improve the training included the use of role play for dental teams to work step-by-step through the process of recruiting and treating ‘mock’ patients with all the appropriate paperwork, the use of videos to demonstrate how to talk to patients about the trial and the provision of scripts to provide dental teams with a form of words to use to describe the interventions.

## Treatments involved in the trial arms

### Dental teams’ experiences

Dental teams’ experiences of the treatment involved in the trial arms came under two sub-themes: preparedness for providing the treatments and their perceptions of parents’ and children’s preferences for treatments.

#### Preparedness for providing the treatments

Despite receiving the training, some dentists did not feel well prepared to provide some of the treatments required, including the Hall Technique, the surgical arm and radiographs. This was either because of a lack of experience, confidence or conviction that the intervention was not possible in general dental practice:

"Dentist I: *‘Most of us in the room wouldn’t have done the Hall Technique with kids....you’ve got to be careful about new techniques ’.*"

"Dentist G: ‘*The only one that I had a doubt about was the full surgical arm, do you really want to be putting a rubber dam, and putting files down Ds and Es when there’s 4 s and 5 s underneath?.’*"

Despite being aware of national clinical guidelines on the use of radiographs, dentists described concerns about patient and parental acceptance, exposure to radiation and diagnostic quality of the radiographs taken. Radiographs were generally not taken in general practice for children less than six years of age.

"Dentist B: *‘They’re just not tolerated by that age group, although you know that you should take them anyway.’*"

"Dentist D*: ‘Sometimes the dentition looks fine to them, and you say, “I’ll try and take some bitewings”, and they are suspicious. You spend twenty minutes explaining, and they go, “oh alright then”.*"

"Dentist G: ‘*Yeah because we’re not massively into taking x-rays of kids’ teeth purely and simply because of the radiation’.*"

"Dentist B: *‘Even if you take them, they’re not clear, unless I take rubbish bitewings.’*"

The use of radiographs was seen as an aspect of clinical practice that differed markedly between specialist pediatric dentistry and dentistry to children in general dental practice.

Recommendations for improving preparedness for delivering the treatment in the trial arms included further training in a local clinical skills laboratory with local clinical staff and encouraging practitioners to refine the techniques on their own patients prior to the actual start of recruitment.

### Dental teams’ perceptions of parent or child preferences

Dental team members described their perceptions of parent or child preferences for allocation to the three arms of the trial and specifically to the use of preformed metal crowns.

Dental teams described how parents responded to the trial, particularly, not knowing until the next visit, the arm of the trial their child would be allocated to. This was felt to have affected recruitment. Informing parents that they can change their minds about participating seemed to have eased their concerns.

"Dentist A: *‘Our patients have had no problem being part of trial but almost every single one has not liked is that they don’t know on the day what they are coming back for.’*"

Some dentists had experienced negative reactions from parents about the appearance of preformed metal crowns despite showing the parents the crowns before placement, other dentists reported no problems among parents. Children seemed to accept the appearance of the crowns.

In general, dental team members seemed able, through discussion, to address most of the parent’s or children’s concerns. However, this did take time and finding an appropriate form of words to explain aspects of the trial to parents and children was difficult for some dentists.

### Parents’ experiences

Parents generally did not know there were so many different approaches for the management of dental caries in children. They were surprised to hear of the different options and some desired more details on what the three arms entailed. Parents’ preferences for the different arms seemed to be related to whether they thought the treatment worked or if their child had any symptoms:

"Parent 4, mother: ‘*They’ve only had that banana polish…I think he’s gonna have to have some more done. That polish doesn’t work on him. It doesn’t work on Zara either’*."

"Parent 3, mother: ‘*He hadn’t had any toothache or anything, if he had, it might have been different, I would maybe have changed’.*"

In contrast to dentists’ perceptions, parents did not mind not knowing which arm their child was allocated to as long as they felt they could change their minds about being involved:

"Parent 6, mother: ‘*I think she had the coating. I was just told that I could say ‘no’ to the treatment, but that the type of treatment she had would be random…I was happy with that.’*"

Improvements could be made in the explanation of the arms including reassurance that there are many different approaches which have all been shown to be effective. Parents’ preferences for the means of communication of these explanations are provided in the second theme, recruitment.

## Trial documentation

Dentists and dental team members commented on both the volume and design of the trial documentation. In terms of volume, while they were aware that copies of documents were required to comply with governance arrangements, they found it difficult to identify the correct documents required for the different stages of the patients’ progress through the trial. This confusion led to mistakes being reported by some. Several practices found their own solution to handling the volumes of paperwork by making up packs containing all the paperwork needed for a patient.

"Dentist B: *‘I don’t fish for all the different ones now because I was finding it was hurting my brain so I find there was just five… I print each one of those off, and that’s easier and put it into each individual pack, it made it so much easier than trying to fish through that huge lever arch file which is full of loads of pieces of paper. I know that we needed them for ethics, but they were intermingled with the things that we needed but also the things that we need legally but you never have to look at ever.’*"

In terms of the design of the documentation, while the supporting documents were felt to be a good idea, modifications from the dental teams were suggested to improve their usefulness.

## Discussion

This study aimed to describe service providers’ and users’ perspectives on the pilot FiCTION trial. Overall service providers, children and parents found the pilot trial to be well conducted and an interesting experience and they were able to provide valuable recommendations to inform the design and conduct of the main FiCTION trial and other trials involving dental practices. These recommendations will now be discussed.

### Recruitment of practices to the main trial

Several factors were identified as important to the recruitment of practices to the main trial. Firstly, the challenging nature of being involved in a trial needs to be acknowledged during recruitment and training of dental practices. Secondly, the process of paying practices for their time to be involved in the trial should be clarified with practitioners before the start of the trial. Thirdly, practitioners should be advised that patients will require longer appointments than normal for involvement in the trial and would prefer appointments out of school time. Hopper and colleagues also described a lack of understanding about the research process, of appropriate remuneration and time pressures as potential barriers to involvement of dentists and team members in prospective research [8]. A factor not previously reported in the dental literature was the recommendation for recruitment of whole practices with participation of all members of the practice team rather than individual practitioners.

### Training and support of dental practices

As a result of the findings of this study, the time between training and commencement of recruitment for the FiCTION main trial will be shorter and will include further training in a local clinical skills laboratory. Practitioners will also be encouraged to practice the techniques on their own patients after the training, before the trial begins. Training of practitioners will include how to deal with common concerns from parents and children including the uncertainty of allocation, existing preferences for specific treatments and the aesthetics of preformed metal crowns. While the intervention preferences of clinicians and patients have previously been identified as serious potential threats to the validity of trials, a systematic review of RCTs found that the effect of treatment preferences could be minimized if a number of approaches to the study design, conduct and analysis were adopted. Such approaches include the wording and format of patient information and quantitative assessment and qualitative exploration of preferences during trials [5]. Previous research on preformed metal crowns found them to be generally viewed favorably by children and their parents although communication and clinical expertise were highlighted as important in ensuring child and parental acceptance [18].

The second main recommendation regarding training and support was the format of the practice trial documentation. All documentation for the FiCTION main trial will be refined with dental team members’ involvement. This approach should be considered as good practice for dental practice-based research more generally.

### Recruitment of patients

Despite each dentist only being expected to recruit a low number of patients during the pilot trial, recruitment proved difficult and failed to achieve the target number. The methods of recruitment of patients for the FiCTION main trial will therefore be changed and will involve less reliance on recruitment through sending parents information packs and more time for dental teams to discuss the trial with patients in person. A study of recruitment processes across a range of trials involving children found face-to-face discussions to be valued more highly by children and parents in decisions about recruitment than participant information leaflets [6].

One area of clinical practice highlighted by the pilot trial which also had significant impact on recruitment of patients was the low number of radiographs prescribed by dentists. Fewer radiographs were taken by general practitioners than anticipated. It is thought that this contributed to the number of eligible participants (that is, those where caries was detected) being lower than expected. Previous research has also found practitioners to prescribe fewer radiographs for primary teeth than specialists [19,20]. The reasons for this appeared to be multi-factorial and changing practitioners’ behavior to increase the number of radiographs taken, in line with national guidelines [21] is likely to take time and be difficult to achieve.

### Improving parental and child engagement in the main trial

Finally, the findings of this study suggest that improvements should be made to the explanations given to children about the trial and also to parents on the effectiveness of the treatments in the trial. In the FiCTION main trial this will be facilitated by the provision of a script for dentists participating in the main study which is developed through involvement of parents and children. Shilling and colleagues reported how involvement of parents and children in trials could be improved and misunderstandings avoided through better communication from practitioners [6]. Child-centered resources including a story book will be developed to improve children’s experiences of the main trial. Improving children’s experiences is important if they are to be allowed to make an active contribution to the research process [22]. Overall, more research is needed into existing barriers to involving patients and the public in dental research and exploration of approaches to improve their involvement.

### Limitations

While qualitative research generally requires small numbers of participants, the limited number of children involved in this study did not allow full exploration of children’s experiences of the different interventions. The main trial will include an in-depth child-centered qualitative component to investigate fully their perspectives on the acceptability of these different treatments.

## Conclusions

This study aimed to describe service providers’ and users’ perspectives on participation in a pilot trial examining the effectiveness of different approaches for the management of dental caries in children. The findings have provided valuable recommendations and are being used to refine the main trial and improve the recruitment of dental practices and patients, training and support for dentists and engagement of children and parents.

## Competing interests

The authors declare that they have no competing interests

## Authors’ contributions

ZM participated in the design of the study, devised the topic guides, conducted some of the interviews and analysis and prepared the first draft of the manuscript. CD recruited dental participants and helped draft the manuscript. MH conducted some of the interviews and the analysis. CS participated in the design of the study, particularly the topic guides and recruited some of the parent/child participants. GD, JC and NI conceived of the idea for the study, participated in its design and helped draft the manuscript. NI helped design the topic guides. HR participated in the design of the study, contributed to the analysis of the results and helped to draft the manuscript. All authors have read and approved the final manuscript.
